# Bacterial sensing and response for neutralization and detoxification of environmental ammonia

**DOI:** 10.1128/jb.00401-25

**Published:** 2026-01-12

**Authors:** Yongsung Kang, Gi-Young Kwak, Yewon Nam, Ingyu Hwang

**Affiliations:** 1 Institute of Agriculture and Life Science, Gyeongsang National University 505651, Jinju, Republic of Korea; 2 Department of Agricultural Biotechnology, Seoul National University 539783, Seoul, Republic of Korea; National Institutes of Health, Bethesda, Maryland, USA

**Keywords:** ammonia, *Burkholderia glumae*, two-component system, sensor kinase, transcriptome

## Abstract

Ammonia present in the environment is a major source of nitrogen, but it can be toxic to bacteria. While the biochemical mechanisms involved in the metabolic detoxification of cellular ammonia are well understood, little is known about how bacteria manage toxic external ammonia to survive, especially when ammonia is present as a waste product at high concentrations. Here, we demonstrate that a two-component system consisting of the sensor kinase GrtK and the response regulator GrtR is responsible for sensing and neutralizing toxic environmental ammonia produced as a waste product by the rice pathogen *Burkholderia glumae*. The growth of null mutants of *grtK* or *grtR* was inhibited in amino acid-rich media such as Luria-Bertani medium, but no growth inhibition was observed in amino acid-free media. The expression of *obcAB*, responsible for the biosynthesis of the previously known neutralizing agent oxalate, was dependent upon external ammonia concentration in a GrtR-dependent manner. Significant changes in fluorescence were observed when cells of *B. glumae* carrying a recombinant plasmid of the modified circular permutation GFP gene fused to *grtK* were incubated with compounds containing ammonium, suggesting that GrtK interacts selectively with external ammonia. Transcriptome analysis of *grtK* and *grtR* mutants also showed that GrtK and GrtR are involved in the metabolic detoxification of cellular ammonia as well. These results indicate that GrtK is an external ammonia sensor that is not a member of the ammonia transporter protein family and works together with the response regulator GrtR to counter the risk posed by its own metabolism.

**IMPORTANCE:**

Excessive accumulation of external ammonia, resulting from the deamination of amino acids used as carbon sources, can be toxic to bacteria. However, there is a limited understanding of how bacteria sense toxic environmental ammonia and how this sensing is translated into outputs that regulate gene expression to avoid toxicity. We found a previously unknown bacterial two-component system composed of GrtK and GrtR responsible for sensing and neutralizing toxic environmental ammonia. Understanding how pathogenic bacteria modify their toxic environment for survival can aid in the development of appropriate treatments and provide drug targets to control pathogens. Our findings suggest that GrtK and GrtR could be potential targets for drug development to control rice panicle blight caused by *B. glumae*.

## INTRODUCTION

When bacteria utilize amino acids as carbon sources, these amino acids are deaminated, resulting in the production of ammonia (1). The excreted ammonia can cause the environment to become alkaline, which can be detrimental to alkaline-sensitive bacteria (2, 3). Therefore, bacteria need to monitor environmental ammonia levels and manage toxic environmental conditions to ensure their growth and survival. While the biochemical and molecular mechanisms involved in the assimilation or detoxification of cellular ammonia are well characterized in bacteria (4, 5), little is known about how bacteria sense and respond to external ammonia to survive in the environment. Ammonia transporters (Amt) have a high affinity for ammonia, suggesting that they may sense external ammonia and regulate its influx (6). Amt proteins are commonly conserved in bacteria, fungi, plants, and animals (7–9). The function of Amt proteins includes external ammonia sensing, in addition to ammonia transport, in *Saccharomyces cerevisiae* and *Rhodobacter capsulatus* (10, 11). In *Escherichia coli*, the Amt protein AmtB binds to GlnK when the intracellular nitrogen concentration reaches a certain level, inhibiting further ammonium transport (12, 13). Therefore, Amt is generally regarded as an ammonia sensor in various organisms, including bacteria. However, direct physical evidence supporting their role in sensing external ammonia is lacking, except in anaerobic ammonium-oxidizing (anammox) bacteria (14, 15).

The process by which bacteria sense environmental signals and transmit them into the cell is commonly controlled by a two-component system (TCS), which consists of sensor kinases and cognate response regulators (16). However, it is not yet known whether TCS is involved in sensing environmental ammonia. To identify the TCS responsible for sensing external ammonia, we used *Burkholderia glumae* BGR1, which causes rice panicle blight as a model organism based on the fact that this bacterium is sensitive to alkaline pH but survives the toxic alkaline environment caused by excreted ammonia upon growth in Luria-Bertani (LB) medium (17). The bacterium produces oxalate in a quorum-sensing (QS)-dependent manner to neutralize the severe alkaline environment (17, 18). Expression of the oxalate biosynthetic genes *obcAB* was dependent upon QsmR (QS master regulator) in *B. glumae* (17, 19). Although the QS-mediated ammonia neutralization process has been well documented regarding how *B. glumae* cells respond to externally accumulated ammonia by producing oxalate, it remains unknown how they sense and respond to toxic external ammonia.

This study identified a previously unknown TCS consisting of *grtK* and *grtR*, which are important for the growth under external ammonia stress and for the expression of *obcAB*. This led us to determine whether GrtK acts as a sensor for external ammonia and GrtR as a response regulator for the expression of *obcAB*. Our findings indicate that GrtK functions as an extrinsic ammonia sensor, and both GrtK and GrtR are involved in regulating oxalate biosynthesis and genes responsible for the metabolic detoxification of cellular ammonia. To the best of our knowledge, GrtK and GrtR represent the first demonstrated environmental ammonia sensor and response regulator in non-anammox bacteria capable of counteracting the toxicity of externally accumulated ammonia.

## RESULTS

### Identification of a TCS consisting of *grtK* and *grtR*

**Fig 1:**
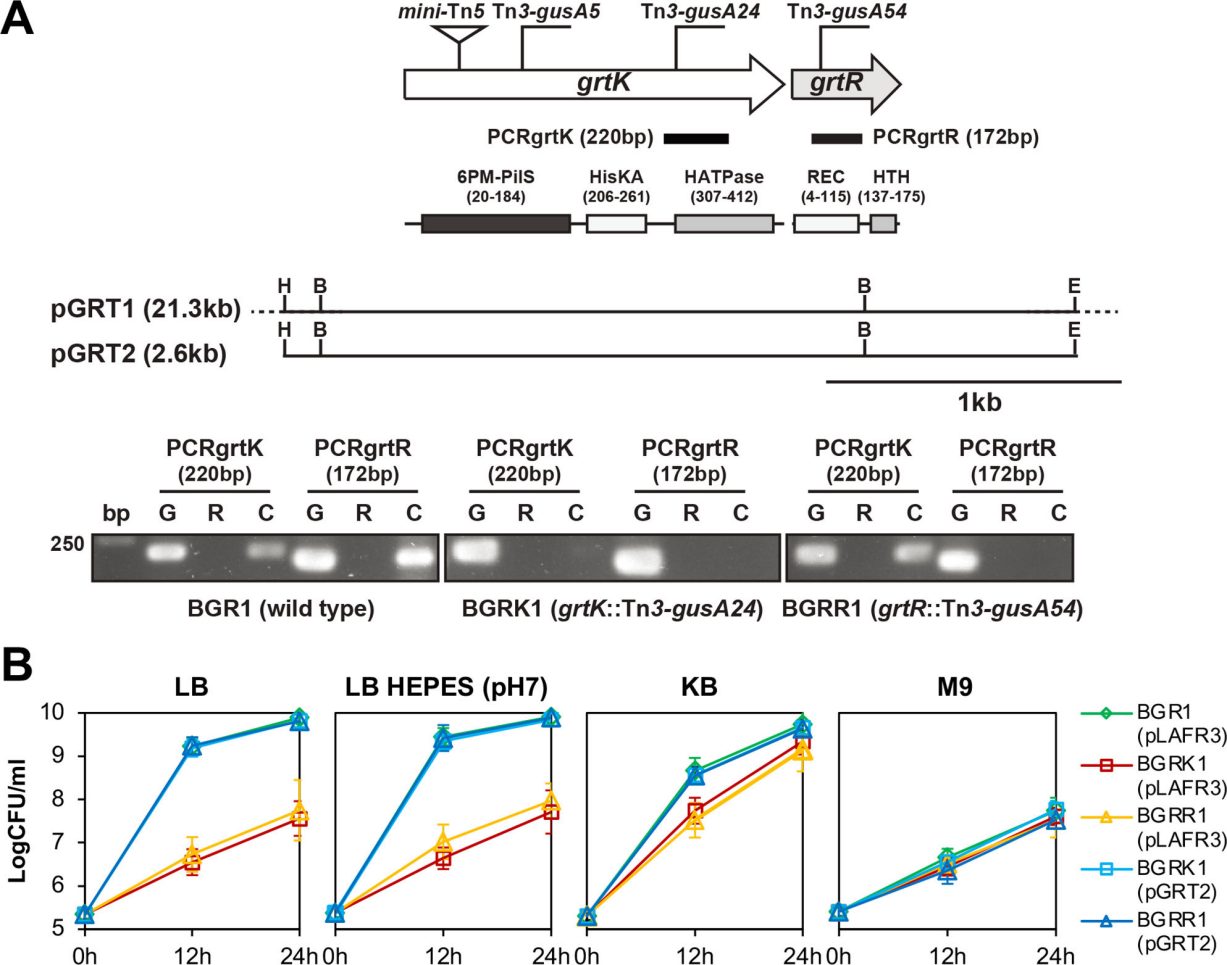
Growth inhibition of *B. gumae* deficient in GrtK/R TCS in amino acid-rich media. (**A**) The genetic organization of the gene locus encoding GrtK/R TCS is presented. Arrows with arrowheads indicate the direction and length of the *grtK* and *grtR* genes. Vertical bars in the map denote the positions and orientations of Tn*3-gusA* insertions. The short, thick bars below the arrows indicate PCR products from the corresponding reverse transcription reactions. The expected sizes of the PCR products are indicated in parentheses beside each numbered PCR. Lane G: PCR products from chromosomal DNA as a template; lane R: PCR products from total RNA; lane C: PCR products from cDNA. GrtK contains a PilS-like transmembrane domain (6PM-PilS; PF25323), a C-terminal HK domain (HisKA; PF06580), and an HK/HSP90-like ATPase domain (HATPase; PF00512). GrtR has an N-terminal response regulatory receiver domain (REC; PF00072), which is phosphorylated by HK, and a Fis-type DNA-binding helix-turn-helix (HTH) domain (HTH; PF02954) in its C-terminal region. The restriction enzyme sites are indicated as follows: E, *Eco*RI; B, *Bam*HI; H, *Hind*III. (**B**) The *B. glumae* wild-type strain BGR1, BGRK1 (BGR1 *grtK*::Tn*3-gusA24*), and BGRR1 (BGR1 *grtR*::Tn*3-gusA54*) with pLAFR3 (empty vector), and the *grtK* and *grtR* mutants complemented with pGRT2, were cultured in LB, HEPES-buffered LB (pH 7), KB, and M9 medium. Bacterial growth was measured as colony-forming units per milliliter (CFU/ml). Error bars indicate the standard error ranges of three independent experiments.

From a pool of random mutants generated using Rescue mini-Tn*5* (Table S1), a growth-defective mutant was identified that did not sustain initial growth in amino acid-rich media such as LB. This mutant was found to have a Rescue mini-Tn*5* insertion in the *grtK* gene (bglu_1g33790; GenBank accession number: ACR30441.1) encoding a putative histidine kinase (HK). The *grtR* gene (bglu_1g33780; GenBank accession number: ACR30440.1), which encodes a putative response regulator, was located downstream of *grtK* (Fig. 1A). To further characterize *grtK* and *grtR*, pGRT1 containing both genes was mutagenized with Tn*3-gusA* followed by marker-exchange into wild-type BGR1. This resulted in BGRK1 (BGR1, *grtK*::Tn*3-gusA24*) and BGRR1 (BGR1, *grtR*::Tn*3-gusA54*), respectively (Fig. 1A). To determine whether *grtK* is co-transcribed with *grtR* in *B. glumae*, reverse transcription-PCR (RT-PCR) analysis was performed using cDNA synthesized from wild-type BGR1 mRNA with specific primers was performed (Table S2). As shown in Fig. 1A, *grtK* and *grtR* were co-transcribed, and the Tn*3-gusA24* insertion in *grtK* had a polar effect on the expression of *grtR*.

Analysis of GrtK, which consists of 427 amino acids, using Pfam/InterPro (https://www.ebi.ac.uk/interpro) revealed a PilS-like transmembrane domain (pfam:PF25323) containing six transmembrane helices, similar to those found in the N-terminus of sensor protein kinases PilS and RegB, and related bacterial proteins. The analysis also revealed a C-terminal HK domain (pfam:PF06580) and an HK/HSP90-like ATPase domain (pfam:PF00512). These two C-terminal domains belong to the sensor HK RisS, a periplasmic cytoplasmic domain superfamily (InterPro entry: IPR038421), and are presumed to be kinase regions that sense the pH of the environment (Fig. 1A). Additionally, Pfam/InterPro analysis of GrtR, which consists of 180 amino acids, revealed that this protein is composed of an N-terminal response regulatory receiver domain and a C-terminal effector domain with DNA-binding activity, similar to most response regulatory proteins. The N-terminal response regulatory receiver domain (Pfam:PF00072) of GrtR belongs to the CheY-like superfamily (InterPro entry:IPR011006) and represents a domain that can be phosphorylated upon receiving a signal from a sensor partner in TCS. The C-terminal DNA-binding domain, a DNA-binding helix-turn-helix (HTH) domain, Fis-type (Pfam:PF02954), encodes an HTH DNA-binding motif at the C-terminus, which shows high similarity to other HTH motifs of regulators such as the nitrogen assimilation regulatory protein (NtrC) found in species such as *Azotobacter*, *Rhodobacter*, and *Rhizobium* (Fig. 1A).

In our analysis of the functional domains of GrtK using the Pfam/InterPro database, we found no Amt modules or significant homology to known ammonium transporters, such as *Ks*-Amt5, which has been documented in the Protein Data Bank (PDB-ID 6EU6). This transporter features an HK directly attached to an N-terminal Amt module that binds exogenous ammonia in the anammox bacterium “*Candidatus* Kuenenia stuttgartiensis.”

### Growth defects of *grtK* and *grtR* mutants in amino acid-rich media

**Fig 2:**
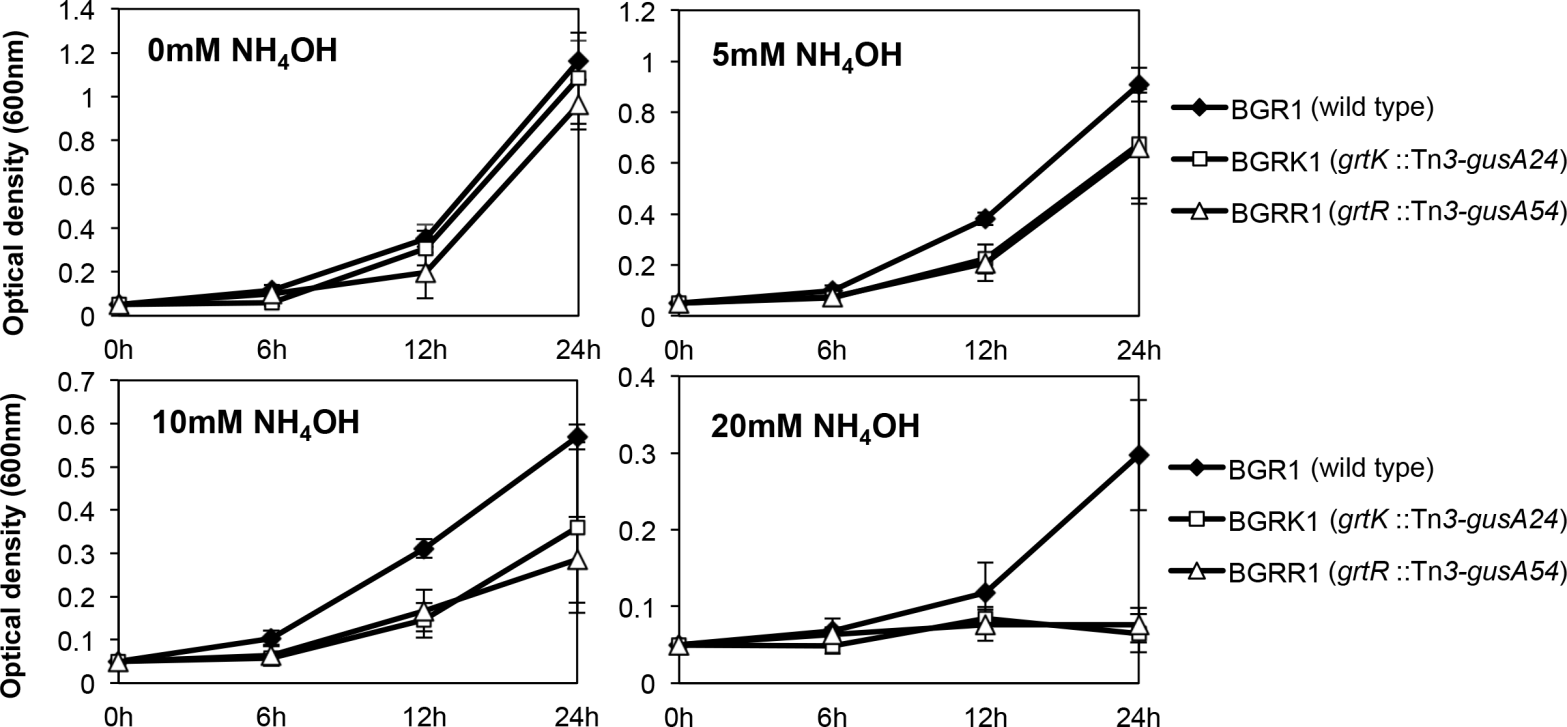
The growth inhibitory effect of *B. glumae* GrtK/R TCS mutants was assessed with respect to external ammonia concentration. *B. glumae* wild-type strain BGR1, BGRK1 (BGR1 *grtK*::Tn*3-gusA24*), and BGRR1 (BGR1 *grtR*::Tn*3-gusA54*) were grown in M9 minimal medium supplemented with various concentrations of NH_4_OH (0–20 mM). Error bars indicate the standard error ranges from three independent experiments.

The growth of the *grtK* and *grtR* mutants, BGRK1 (BGR1, *grtK*::Tn*3-gusA24*) and BGRR1 (BGR1, *grtR*::Tn*3-gusA54*), was significantly slower than that of the wild type in LB broth or LB broth supplemented with 100 mM HEPES (4-[2-hydroxyethyl] piperazine-1-ethanesulfonic acid), pH 7.0 (Fig. 1B). However, compared to the inhibition observed in LB medium, the growth inhibition was less noticeable in King’s B (KB) medium, which contains fewer amino acids than LB medium, and no growth difference was observed in M9 minimal medium (Fig. 1B). When the wild type was cultivated in LB broth, significant accumulation of external ammonia was observed, whereas a relatively lower level of ammonia accumulated in KB medium (Fig. S1). In LB medium, the *grtK* and *grtR* mutants exhibited less pronounced accumulation of external ammonia compared to the wild type, which can be attributed to growth inhibition (Fig. S1). No increase in external ammonia accumulation was detected in the M9 minimal medium (Fig. S1). Upon the addition of 5, 10, or 20 mM of ammonium hydroxide to M9 minimal medium, the growth of *grtK* and *grtR* mutants was inhibited in a concentration-dependent manner, while the wild type exhibited normal growth (Fig. 2). These results indicate that the *grtK* and *grtR* mutants are sensitive to external ammonia.

### GrtK and GrtR regulate oxalate biosynthesis independent of QS

**Fig 3:**
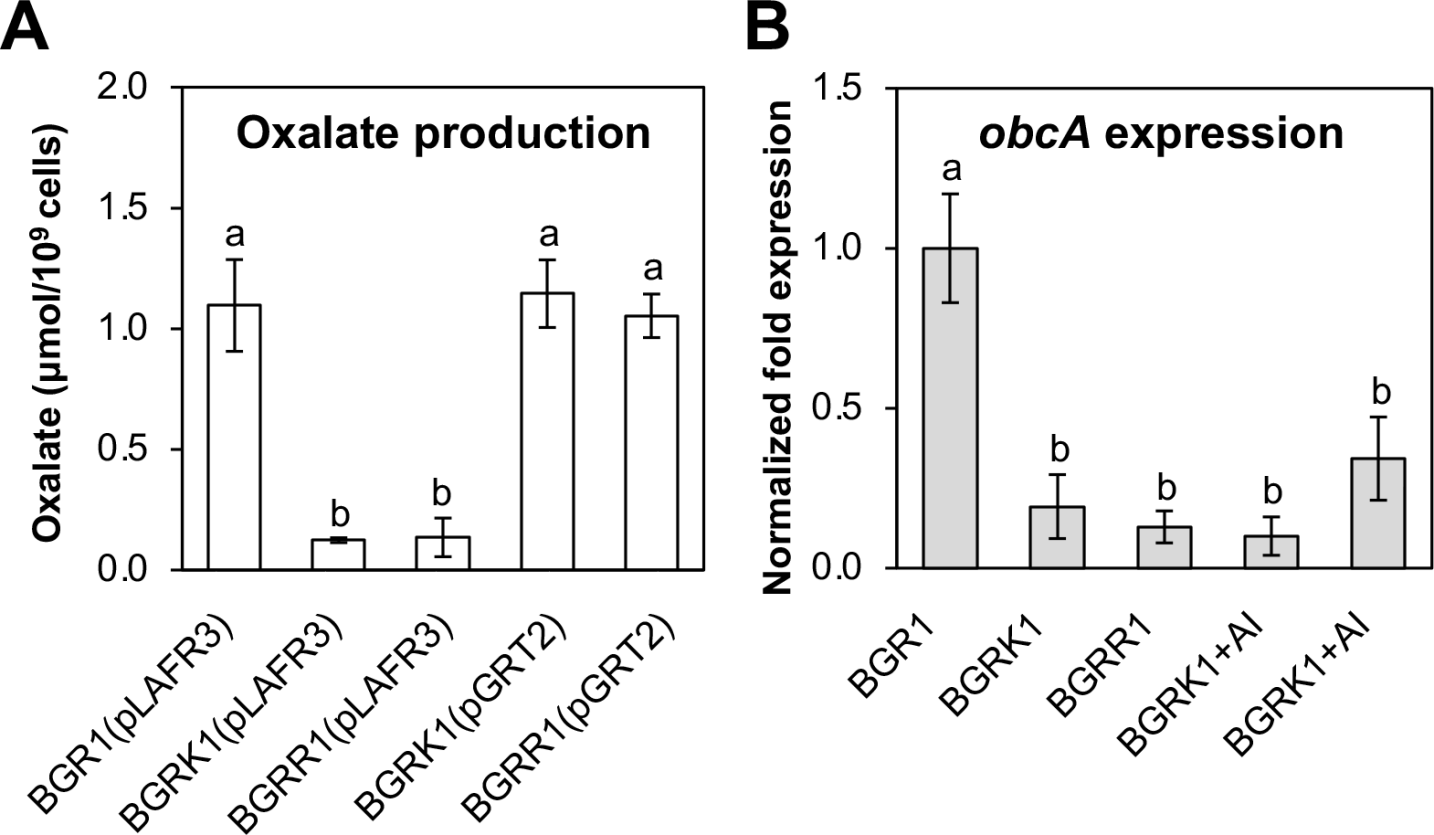
Oxalate production and the expression level of the oxalate biosynthesis gene (*obcA*) were examined in GrtK/R TCS mutants. (**A**) Oxalate production was measured in the wild type, strain BGR1, BGRK1 (BGR1 *grtK*::Tn*3-gusA24*), and BGRR1 (BGR1 *grtR*::Tn*3-gusA54*) in LB medium using an oxalate assay kit with three biological replicates. (**B**) The *obcA* gene expression levels were quantified in the wild-type strain BGR1, BGRK1 (BGR1 *grtK*::Tn*3-gusA24*), and BGRR1 (BGR1 *grtR*::Tn*3-gusA54*) in LB medium. Gene expression in the mutant strains was also measured with the addition of 1 μM of C8-HSL autoinducer (AI). The bars represent ±SE, and the letters (a and b) above each mean indicate groups with statistically significant differences (*P* < 0.05) in oxalate production and *obcA* gene expression, as determined by analysis of variance/Tukey correction for multiple comparisons.

Since oxalate neutralizes ammonia-mediated alkaline conditions in *B. glumae* BGR1, we investigated whether mutations in *grtK* and *grtR* affect oxalate production. The *grtK*::Tn*3-gusA24* and *grtR*::Tn*3-gusA54* mutants produced significantly lower levels of oxalate than the wild type, but oxalate production was fully recovered by providing *grtK* and *grtR in trans* in a multi-copy number plasmid pGRT2 (Fig. 3A). We then investigated whether GrtK and GrtR are involved in the expression of *obcA* by quantitative RT-PCR (qRT-PCR) analysis. The expression of *obcA* was significantly reduced in the *grtK* and *grtR* mutants (Fig. 3B). The addition of 1 μM *N*-octanoyl homoserine lactone (C8-HSL), a QS signal of *B. glumae*, to the culture of the *grtK* and *grtR* mutants did not change the expression level of *obcA* (Fig. 3B). To determine whether GrtK and GrtR affect the expression of *qsmR*, we estimated *qsmR* expression levels by qRT-PCR analysis. There was no difference in *qsmR* expression levels between the wild type and the *grtK* and *grtR* mutants (Fig. S2). These results indicate that GrtK and GrtR control the expression of *obcA* independently of QS.

### Selective specificity of GrtK to environmental ammonia

**Fig 4:**
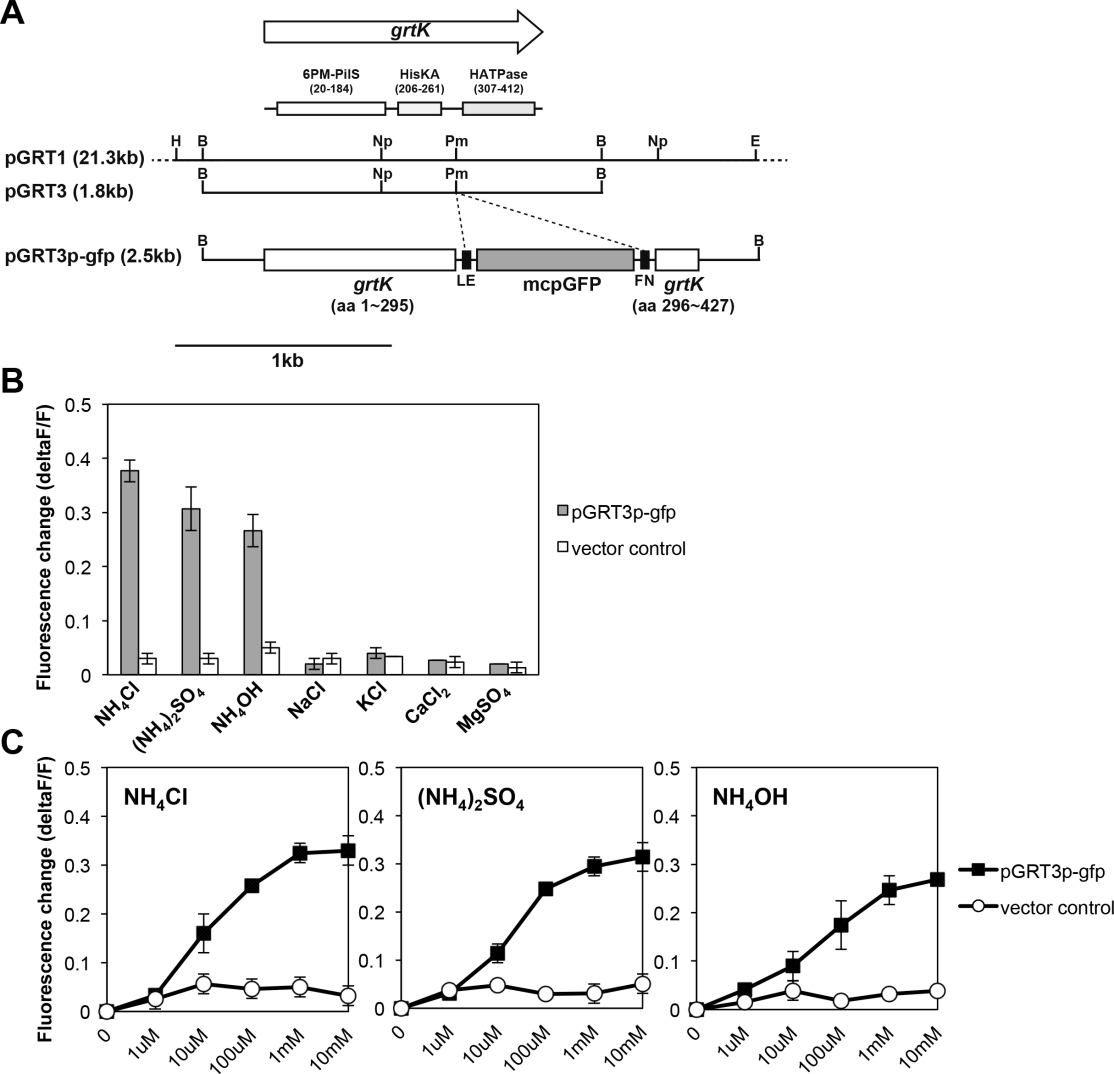
The construction of genes encoding the recombinant GrtK-mcpGFP fusion protein and the ammonia specificity of the sensor kinase GrtK are presented. (**A**) Arrows with arrowheads represent the direction and length of the *grtK* gene encoding GrtK. GrtK contains a PilS-like transmembrane domain (6PM-PilS; PF25323), a C-terminal HK domain (HisKA; PF06580), and a HK/HSP90-like ATPase domain (HATPase; PF00512). The mcpGFP gene was fused between the region coding the C-terminal HK domain and the ATP-binding subdomain of *grtK* to construct pGRT3p-gfp. The restriction enzyme sites are indicated as follows: E, *Eco*RI; B, *Bam*HI; H, *Hind*III; Np, *Nsp*I; Pm, *Pma*CI. (**B**) *B. glumae* wild-type strain cells expressing the GrtK-mcpGFP fusion protein were treated with the indicated chemicals at a concentration of 1 mM. (**C**) *B. glumae* wild-type cells expressing the GrtK-mcpGFP fusion protein were treated with various concentrations of compounds with ammonium (NH_4_) molecules (0–10 mM). Data were normalized to a water-treated control. Error bars indicate the SE ranges of three independent experiments, and only data for the ammonium compounds treatments were significantly different from the control (*P* < 0.05) determined using analysis of variance/Tukey’s test.

To determine whether GrtK functions as a sensor of environmental ammonia, the modified circular permutation GFP (mcpGFP) gene was fused between the region coding for the C-terminal HK domain and the HK/HSP90-like ATPase domain of *grtK* to construct pGRT3p-gfp (Fig. 4A). The purpose of this gene fusion was to monitor the protein structural changes after the N-terminal portion of GrtK binds to ammonia using fluorescence-based cell biological methods. Fluorescence changes (ΔFluorescence/Fluorescence) of *B. glumae* BGR1 carrying pGRT3p-gfp were monitored during growth by challenging it with various compounds. Significant fluorescence changes were observed with compounds containing ammonium molecules such as NH_4_Cl, (NH_4_)_2_SO_4_, and NH_4_OH (Fig. 4B). These changes were dependent on the concentrations of NH_4_Cl, (NH_4_)_2_SO_4_, and NH_4_OH (Fig. 4C). These results demonstrate that GrtK is a sensor of environmental ammonia.

### GrtK and GrtR affect the metabolic detoxification of cellular ammonia

**Fig 5:**
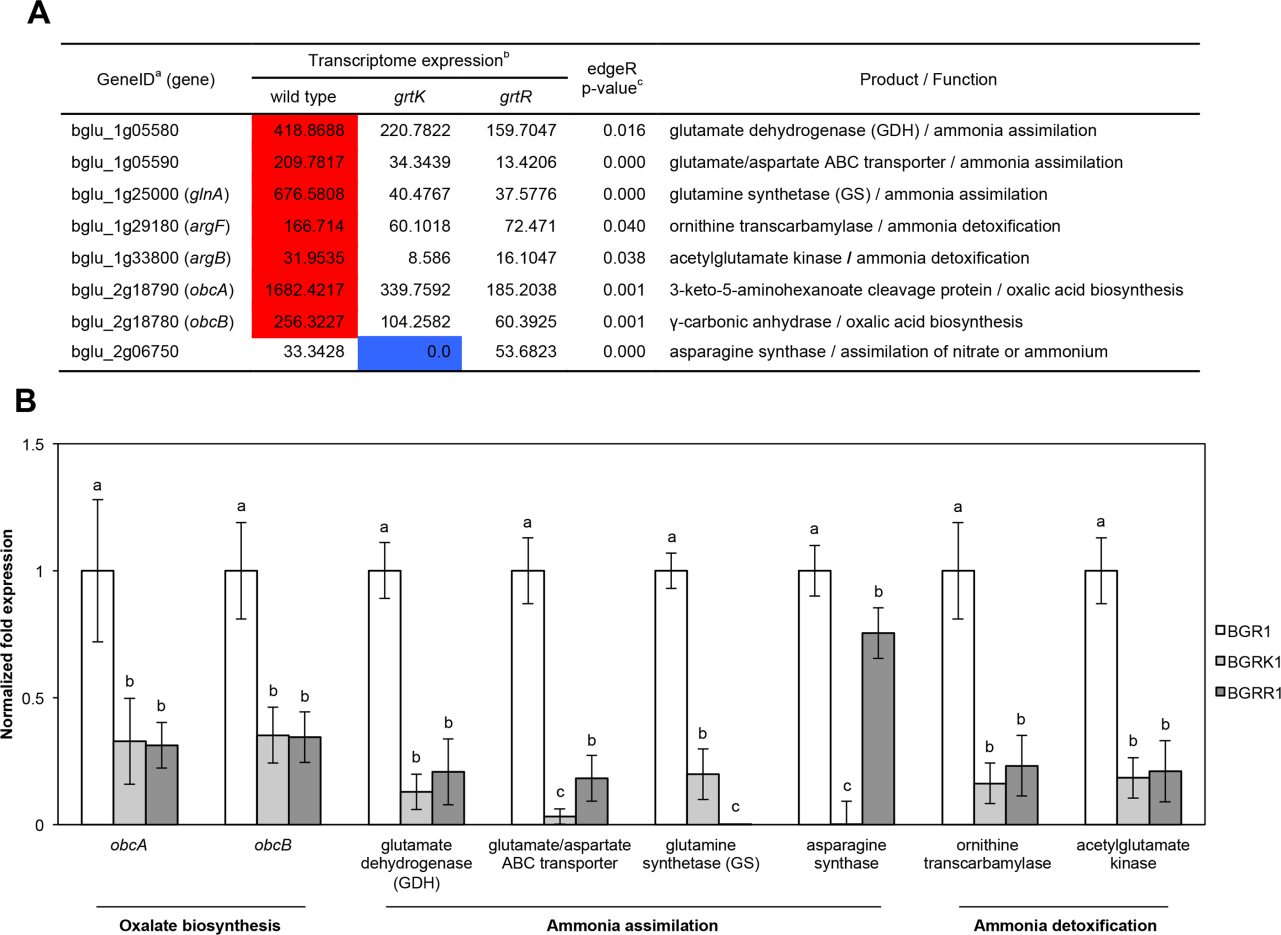
Key genes involved in ammonia neutralization and detoxification showed differential expression in *grtK* or *grtR* mutants. (**A**) Gene IDs (a) were obtained from the *B. glumae* BGR1 genome database (GenBank accession numbers: CP001503–CP001508). Transcriptome expression (b) is represented as reads per kilobase per million mapped reads (RPKM). RPKM = gene mapped reads/[total mapped reads (millions) × gene length (Kb)]. Differential gene expression analysis (c) was based on the empirical Bayes estimation (edgeR). Genes shown in the table were selected based on a *P*-value of ≤0.05. (**B**) Gene expression levels were quantified in the wild-type strain BGR1, BGRK1 (BGR1 *grtK*::Tn*3-gusA24*), and BGRR1 (BGR1 *grtR*::Tn*3-gusA54*) in LB medium after 12 h of incubation via qRT-PCR with three biological replicates. The letters (a, b, and c) above each mean indicate groups with statistically significant differences (*P* < 0.05) in gene expression levels, as determined by analysis of variance/Tukey correction for multiple comparisons.

**Fig 6:**
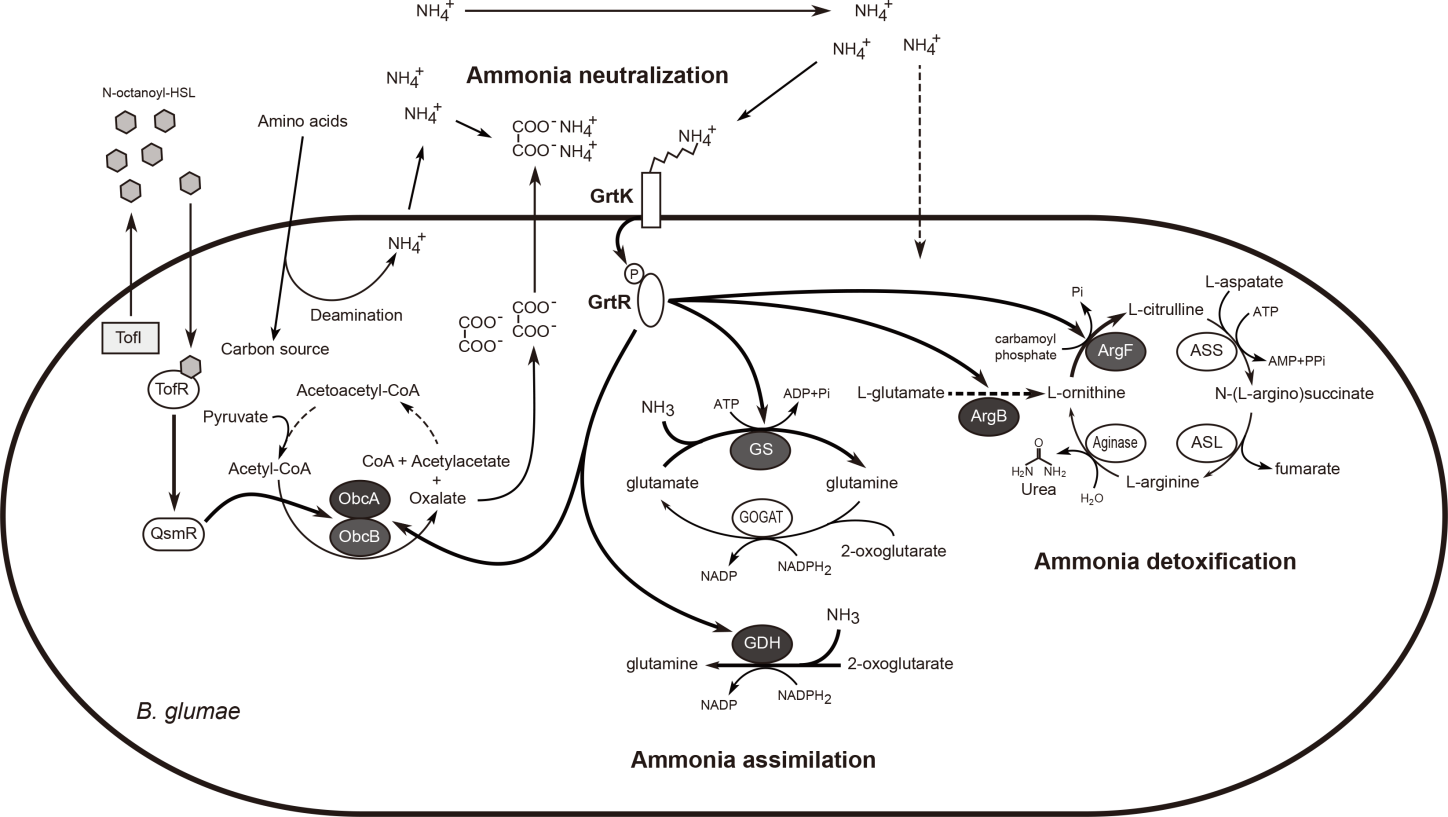
GrtK/R mediates the regulation of genes responding to accumulated environmental ammonia in *B. glumae*. ObcA, 3-keto-5-aminohexanoate cleavage protein for oxalate biosynthesis; ObcB, γ-carbonic anhydrase for oxalate biosynthesis; GOGAT, glutamine oxoglutarate aminotransferase; GDH, glutamate dehydrogenase; ArgB, acetylglutamate kinase; ArgF, ornithine transcarbamylase; ASS, argininosuccinate synthase; ASL, argininosuccinate lyase. Dashed arrows indicate multiple pathways, and bold arrows indicate activation.

To determine whether GrtK and GrtR are involved in metabolic detoxification of cellular ammonia, wild-type BGR1, BGRK1 (BGR1, *grtK*::Tn*3-gusA24*), and BGRR1 (BGR1, *grtR*::Tn*3-gusA54*) were cultured in LB medium at 37°C for 12 h, after which total RNA was extracted and subjected to RNAseq analysis. A full list of differentially expressed genes (DEGs) with *P*-values less than 0.05 in the filter (edgeR) is provided in NCBI’s Gene Expression Omnibus (GEO) database (GEO series number GSE309868). The selected genes were then grouped on the basis of function and homology and summarized according to the expression patterns in wild type and the mutants (Table S3). Significant changes in the *grtK* or *grtR* mutants were observed in the expression of genes responsible for oxalate biosynthesis genes (bglu_2g18790; *obcA* and bglu_2g18780; *obcB*), ammonia assimilation (bglu_1g25000; glutamine synthetase [GS], bglu_1g05580; glutamate dehydrogenase, and bglu_1g05590; glutamate/aspartate ABC transporter), ammonia detoxification (bglu_1g29180; ornithine transcarbamylase and bglu_1g33800; acetylglutamate kinase), nitrogen compound recycling (bglu_2g06750; asparagine synthase), cellular energy production, and the synthesis and metabolism of various amino acids, carbohydrates, and fatty acids (Tables S3 and S4). The expression patterns of genes related to ammonia neutralization, ammonia assimilation, and detoxification were re-examined by qRT-PCR, and the results were consistent with the RNAseq data (Fig. 5A and B). Based on the results of this study, a schematic diagram illustrating the roles of GrtK and GrtR in the neutralization of external ammonia, ammonia assimilation, and ammonia detoxification is presented (Fig. 6).

In contrast to the roles of GrtK and GrtR as positive regulators, certain gene groups are negatively controlled by them. In particular, the expression of genes encoding molecular chaperones or enzymes that respond to elevated cellular stress, such as *dnaK* (bglu_1g06340), *htpG* (bglu_1g26820), *groES* (bglu_2g19340), and superoxide dismutase (bglu_1g29070), is repressed by GrtK and GrtR (Table S4). Additionally, genes encoding RpoH (σ^32^ bglu_1g31450) and the RpoN (σ^54^)-dependent transcriptional regulator (bglu_1p1190) are also negatively controlled by GrtK and GrtR (Table S4).

## DISCUSSION

While ammonia is an essential nitrogen source for bacteria, fungi, and plants, it becomes cytotoxic at high concentrations. Therefore, organisms require significant biological selectivity to detoxify or control its toxicity while up-taking it for nutritional needs. Under nitrogen-limiting conditions, Amt proteins mediate the uptake of ammonia/ammonium into the cells of numerous organisms (7–9). It has been suggested that selective ammonia uptake in bacteria occurs through allosteric regulation of the P_II_ protein complex, which includes Amt proteins (11, 12). However, the experimentally validated mechanism for the Amt-P_II_ complex’s selectivity for ammonia relies on P_II_’s role in detecting the cellular nitrogen status in bacteria (10), leading to controversy regarding whether Amt functions as a “sensor” for external ammonia. On the other hand, unlike the Amt protein of ammonia-assimilating bacteria, *Ks*-Amt5 has an ammonium-binding site within its Amt module, allowing it to function as an ammonium sensor instead of transporting ammonia directly in the anammox bacterium *C*. Kuenenia stuttgartiensis (15). These results suggest that the signal transduction system comprises HK (*Ks*-Amt5) and a response regulator that is yet to be identified (15). Apart from the Amt-P_II_ protein complex, these components are involved in sensing ammonia and transmitting its signal into the cell in anammox bacteria (15). This led us to explore sensors and response regulators for external ammonia, based on our key observation that the *grtK* mutant of *B. glumae*, a non-anammox bacterium, showed growth defects in amino acid-rich media. Consequently, we found that *B. glumae*’s GrtK/R system is a new type of system that senses and signals ammonia independently of Amt.

The *B. glumae* QS mutant grew faster than the wild type because of the metabolic slowing function of QS, but its growth was inhibited due to alkaline toxicity (18). In contrast, the growth defects of *grtK* and *grtR* mutants were not due to alkaline environmental conditions, as adding 100 mM Hepes to the LB broth during growth did not alleviate the mutant’s slow growth. This result suggests that *B. glumae* must have ammonia-sensing and response processes for survival. Thus, we hypothesized that GrtK could be a sensor for extrinsic ammonia in *B. glumae*. We first attempted to prove that ammonia actually binds to GrtK *in vitro*, but despite many efforts, we were unsuccessful because we could not express *grtK* in a heterologous system using the *E. coli* T7 protein expression system. Instead, we used cell biological methods that have been used in plants and yeasts to verify ammonium transceptors (a method that detects dual function transporter and receptor) (20). The basic principle of fluorescence-based cell biology methods is the monitoring of conformational changes following ammonia binding (21). With this method, exclusive binding of ammonia to GrtK was verified (Fig. 4B). However, the absence of an Amt module in GrtK suggested that there must be a unique protein domain or amino acid motif for recognizing ammonia, which should be characterized in future studies. Since GrtK is one of the TCS present in *B. glumae*, we believe that GrtK is not involved in ammonia transport.

In addition to the QS-dependent expression of *obcAB*, we identified another regulatory layer, GrtK and GrtR, that controls oxalate biosynthesis in *B. glumae*. This discovery explains how cooperative cells in *B. glumae* recognize and respond to toxic external ammonia for survival. This mechanism illustrates how bacteria coordinate environmental and density sensing to control gene expression, allowing for better adaptation to changing environments. A remaining question is whether temporal control exists for each regulatory system to efficiently manage *obcAB* expression.

According to an unbiased transcriptional analysis of *grtK* and *grtR* mutants (Fig. S3), the expression of *amtB* (bglu_1g31870; Ammonium transporter of *B. glumae* BGR1) and the P_II_ protein gene (bglu_1g12700; P_II_ protein gene of *B. glumae* BGR1) does not appear to rely on GrtK/R. These results suggest that GrtK/R functions as a unique environmental ammonia-sensing and regulation system, independent of ammonia transport and distinct from the previously established P_II_ protein-mediated cellular nitrogen-sensing and regulatory systems in bacteria.

GrtK/R-dependent genes, identified through unbiased transcriptional analysis of *grtK* and *grtR* mutants, are broadly organized into categories such as energy (ATP) production, amino acid metabolism, and fatty acid metabolism, as well as ammonia assimilation and detoxification (Table S3). It is plausible that the GrtK/R TCS continuously and positively influences a range of processes that activate energy metabolism, promote amino acid and fatty acid metabolism, and eliminate waste products, such as ammonia, that are inevitably generated during these processes.

These results support the hypothesis that the GrtK/R TCS detects high levels of external ammonia during nitrogen-rich growth and consistently regulates metabolic processes to alleviate excessive internal and external ammonia levels. This contrasts with regulatory processes involving Amt, which upregulates Amt expression to increase ammonia uptake under nitrogen-limited conditions (22), and the anammox bacterium *C*. Kuenenia stuttgartiensis, which maintains intracellular ammonium concentration by repressing GS/glutamine oxoglutarate aminotransferase genes when *Ks*-Amt5 expression increases during ammonium-limited growth (23). Therefore, unlike ammonia sensing related to Amt, which is mainly required for obtaining ammonia or ammonium under nitrogen-depleted conditions, the exogenous ammonia monitoring by GrtK/R TCS offers new insights into ammonia-sensing mechanisms that bacteria need to manage toxic waste product risks while maintaining high growth rates under nitrogen-rich conditions.

## MATERIALS AND METHODS

### Bacterial strains and growth conditions

The bacterial strains and plasmids used in this work are listed in Table S1. Strains of *B. glumae* and *E. coli* were grown at 37°C in LB medium (1% [wt/vol] tryptone and 0.5% [wt/vol] of yeast extract; USB Corp., Cleveland, OH, USA) or in LB buffered with 100 mM HEPES (pH 7.0). For experimental purposes, *B. glumae* strains were also cultured on KB medium (1% [wt/vol] proteose peptone [Difco, Franklin Lakes, NJ, USA], 0.15% [wt/vol] of K_2_HPO_4_ and MgSO_4_•7H_2_O, and 1.5% [vol/vol] glycerol) or M9 minimal medium (33.7 mM Na_2_HPO_4_, 22 mM KH_2_PO_4_, 8.55 mM NaCl, 9.35 mM NH_4_Cl, 1 mM MgSO_4_, and 0.3 mM CaCl_2_) with 0.2% [wt/vol] glucose added as a carbon source.

### Rescue *mini*-Tn*5*, Tn*3-gusA*, and marker-exchange mutagenesis

Random mutations were generated in *B. glumae* BGR1 using *E. coli* S17-1 (pRescue *mini*-Tn*5*), as previously described (24). Mutants were isolated by selection on LB agar containing kanamycin. To construct GrtK and GrtR mutants, Tn*3-gusA* was inserted into the *grtK* or *grtR* gene of the pGRT1 plasmid. The insertion site and orientation of Tn*3-gusA* in each mutant were determined by direct sequencing of the plasmid using the primer Tn3gus (5′-CCGGTCATCTGAGACCATTAAAAGA-3′) (25). The mutagenized plasmids with Tn*3-gusA* insertions were individually introduced into the parent strain BGR1, followed by marker exchange into wild-type BGR1 (26). All marker exchanges were confirmed by Southern hybridization analysis (Fig. S5).

### Measurement of ammonia concentration

A gas-sensing ammonia ion-selective electrode (Thermo Scientific, Beverly, MA, USA) was utilized to detect the gas phase of ammonia following the addition of the alkaline reagent to the culture medium. This ammonia electrode employs a hydrophobic gas-permeable membrane to separate the sample solution from the electrode filling solution. Once ammonia dissolves in the sample solution, it diffuses through the membrane until the partial pressure of ammonia is equal on both sides. At this point, the electrode measures the partial pressure in proportion to the ammonia concentration within the sample.

### Quantitative determination of oxalate production

Oxalate levels were quantified using an oxalate assay kit (Libios, Pontcharra sur Turdine, France) according to the manufacturer’s protocol (27). Oxalate oxidase was used to transform oxalate into carbon dioxide and hydrogen peroxide, which then reacted with 3-(dimethylamino) benzoic acid to produce a blue-colored compound. The concentration of this compound was determined by measuring absorbance at 590 nm.

### RNA isolation and qRT-PCR

Total RNA was isolated from *B. glumae* wild-type BGR1, the *grtK* mutant (BGR1 *grtK*::Tn*3-gus24*), and the *grtR* mutant (BGR1 *grtR*::Tn*3-gus54*), using the RNeasy Mini Kit (Qiagen, Venlo, Netherlands) according to the manufacturer’s instructions, with cells cultured in LB or M9 media at 37°C. To remove genomic DNA completely, the isolated RNA was treated with DNaseI (Ambion, Austin, TX, USA) for 1 h at 37°C according to the supplier’s instructions. The primer pairs used in this study for qRT-PCR are listed in Table S2. qRT-PCR analysis was performed to compare transcriptional levels using SsoFast EvaGreen Supermix (Bio-Rad, Hercules, CA, USA) and the CFX96 Real-Time PCR system (Bio-Rad). The thermal cycling parameters were 95°C for 30 s, followed by 35 cycles of 95°C for 5 s and 60°C for 5 s. PCRs were repeated three times, and the fold change in the target genes was normalized to 16S rRNA reference genes using CFX Manager Software (Bio-Rad).

### Construct the recombinant GrtK-mcpGFP fusion protein

To investigate whether the GrtK protein functions as a sensor through interaction with ammonia molecules, we constructed a plasmid, pGRT3p-gfp, containing the mcpGFP gene fused to the C-terminus of the *grtK* gene. The mcpGFP gene (GenBank accession number ADJ53338.1), which includes synthetic linker segments (LE linker: CTCGAG; FN linker: TTTTAA), was synthesized (Fig. S4). The corresponding mcpGFP moiety was amplified using the primer pair PmaCI-F/PmaCI-R (Table S2), which contains the *PmaC*I restriction site (CACGTG). The purified amplified fragment was then digested with PmaCI (New England Biolabs) and ligated into PmaCI-digested pGRT3 containing the *grtK* gene, resulting in pGRT3p-gfp, which encodes a protein in which mcpGFP is fused with a synthetic linker at the 296 amino acid position of GrtK (Fig. 4A).

### Fluorometric analysis for the recombinant GrtK-mcpGFP

Fluorimetric analyses were performed, modifying the method described in the previous study (20). Bacterial cultures were grown overnight in LB broth at 37°C and diluted to OD_600 nm_ = 0.05. After 14 h subculture, 2 mL of cells from each culture were harvested by centrifugation (10,000 × *g* for 2 min), washed twice with 1 mL of DPBS buffer, pH 7.0, and resuspended to OD_600 nm_ = 0.5 in DPBS buffer. To measure the fluorescence response to the addition of the tested chemicals, 50 µL of each chemical (dissolved in water as a 10× stock solution) was added to 200 µL of cells in a 96-well plate (Thermo Scientific, Beverly, MA, USA). Fluorescence was measured at an emission wavelength of 513 nm using an excitation wavelength of 488 nm by a PerkinElmer Victor X3 microplate reader (PerkinElmer, Waltham, MA, USA). The response to compound data is presented as fluorescence changes {(*F*_water_ – *F*_compound_)/*F*_compound_}.

### RNA library preparation and sequencing

Total RNA was extracted from *B. glumae* BGR1, BGRK1 (BGR1 *grtK*::Tn*3-gus24*), and BGRR1 (BGR1 *grtR*::Tn*3-gus54*) grown in LB medium at 37°C for 12 h after subculture, using RNeasy minikits (Qiagen) following the manufacturer’s protocols. The extracted total RNA was treated with RNase-free DNase I (Ambion) to remove DNA. The quantity and quality of the total RNA were evaluated using RNA electropherograms (Agilent 2100 Bioanalyzer, Santa Clara, CA) and by assessing the RNA integrity number (RIN). The NEBNext rRNA Depletion Kit (Bacteria; New England BioLabs, UK) was used for ribosomal RNA depletion according to the manufacturer’s instructions. Libraries for Illumina sequencing were made with the TruSeq stranded mRNA library prep (Illumina, USA) following the manufacturer’s protocol. RNA sequencing was performed on the Illumina NovaSeq6000 platform using paired-end 150 bp sequencing. Image analysis was performed using the NovaSeq6000 control software version 1.3.1, and the output base calling data were de-multiplexed with bcl2fastq version v2.20.0.422, generating fastQC files.

### RNA-seq data analysis

Sequence data for the reference genome was obtained from the NCBI database. Quality-filtered reads were aligned to the reference genome sequence using Bowtie2 (28). Relative transcript abundance was measured in fragments as reads per kilobase of exon sequence per million mapped sequence reads (29). The eggNOG (evolutionary genealogy of genes: Non-supervised Orthologous Groups) database (30) was used to cluster genes into functionally related groups and the Kyoto Encyclopedia of Genes and Genomes database (31) was used to analyze metabolic pathways. Differential gene expression analysis was performed using the empirical Bayes estimation (edgeR) (32). The CLRNASeqTM program (CJ Bioscience, South Korea) was used to visualize mapping results and DEGs.

### Statistical analysis

All statistical analyses and analysis of variance testing followed by Tukey’s honest significance difference post hoc analysis were performed using IBM SPSS Statistics software (ver. 20 x86-x64; IBM Corp., Armonk, NY, USA).

## Data Availability

RNA sequence data for genes showing differential expression in *grtK* and *grtR* mutants compared to the wild type has been deposited in NCBI's Gene Expression Omnibus (GEO) database under series number GSE309868. The authors declare that all relevant data supporting the findings of this study are included in this paper and its supplementary materials.

## SUPPLEMENTAL MATERIAL

Supplemental tables and figures jb.00401-25-s0001.docx Tables S1 to S4 and Figures S1 to S5.

ASM does not own the copyrights to Supplemental Material that may be linked to, or accessed through, an article. The authors have granted ASM a non-exclusive, world-wide license to publish the Supplemental Material files. Please contact the corresponding author directly for reuse.
