# Supplementary material for: Bacterial sensing and response for neutralization and detoxification of environmental ammonia
Source: J Bacteriol. 2026 Jan 12;208(2):e00401-25. doi: 10.1128/jb.00401-25 (PMC12918730; doi:10.1128/jb.00401-25)
Supplement: Supplemental tables and figures — Tables S1 to S4 and Figures S1 to S5. [file jb.00401-25-s0001.docx]

**Table S1.** Bacteria strains and plasmids used in this study

| Strain or plasmid | Genotype or phenotype^a^ | Reference or source |
| --- | --- | --- |
| *Escherichia coli* | | |
| DH5α | F^-^Φ80d*lac*ZΔ*M15*Δ(*lac*ZYA-*argF*) *U169endA1 recA1hsd1hsdR17*(r_k_^-^m_k_^+^) *deoRthi*-1*supE44*λ^-^*gyrA96 relA1* | Gibco BRL |
| HB101 | F^-^*mcrBmrrhsdS20*(r_B_^-^m_B_^-^)*recA13leuB6ara*-*14* *proA2lacY1galK2xyl-5 mtl*-*1* *rpsL20*(Sm^r^) *supE44*λ^-^ | Gibco BRL |
| S17-1 | Tp^r^ Sm^r^ *recA*, *thi*, *pro*, *hsdR*^-^*M*^+^ RP4::2-Tc::Mu:Km ::Tn*7* | (1) |
| *Burkholderia glumae* | | |
| BGR1 | Wild type, Rif^r^ | (2) |
| BGS9 | BGR1 *qsmR*::Ω | (3) |
| BGRK1 | BGR1 *grtK*::Tn*3-gusA24* | This study |
| BGRR1 | BGR1 *grtR*::Tn*3-gusA54* | This study |
| Plasmids | | |
| pBluescript II SK(+) | Cloning vehicle; phagemid, pUC derivative, Amp^r^ | Stratagene |
| pLAFR3 | Tra^-^, Mob^+^ RK2 replicon, Tet^r^ | (4) |
| pRK415 | Mob^+^, *lacZ*, Tet^r^ | (5) |
| pRescue-*mini*Tn*5* | Tn*5*-based transposon vector with an *E. coli* replication site; Km^r^ | (6) |
| pGRT1 | 21.3 kb DNA fragment harboring *grtK and grtR* from BGR1 cloned into pLAFR3 | This study |
| pGRT2 | 2.3 kb *Hind*III-*Eco*R1 DNA fragment harboring *grtK and grtR* from BGR1 cloned into pLAFR3 | This study |
| pGRT3 | 1.8 kb *Bam*HI DNA fragment harboring *grtK* from pGRT1 cloned into pLAFR3 | This study |
| pGRT3p-gfp | 2.5 kb *Bam*HI DNA fragment harbouring mcpGFP gene was fuse to the C-terminus of the *grtK* with synthetic linkers cloned into pRK415 | This study |

Rif^r^, rifampicin resistance; Tet^r^, tetracycline resistance; Km^r^, Kanamycin resistance; Amp^r^; ampicillin resistance

**Table S2.** List of primers used for PCR and qRT-PCR in this study. All the primers used were purchased from Macrogen, Korea.

| Primers | Sequence (5’ to 3’) |
| --- | --- |
| PCRgrtK-F | CAGATCCTCACGATCCTGCT |
| PCRgrtK-R | CGCTGAACGCCAGATACAG |
| PCRgrtR-F | CAGTTCATCACCGTCGACCT |
| PCRgrtR-R | CCGGCTTCGACAGATAGTTG |
| obcA-F | CATGAACGGCCCTCTCTCTA |
| obcA-R | CGAAACATTCGGCGACTTAT |
| obcB-F | ACTACAGCCTGGTCGGCATT |
| obcB-R | CTGGTCGGCATAGAACTCG |
| qsmR-F | GAAATGAGGGAGACCAGTCTGTCTATT |
| qsmR-R | GTTTGCGGTTCCGGGTTATTCATGTTCGATCF |
| GDH-F | CCTGGATGTCGGTCAAGAAT |
| GDH-R | CCCTGGTTCATCGAGTAGGT |
| GluTransport-F | GACCAGAACCAGCAGGTGA |
| GluTransport-R | GTGCCGATCACGAAATAGGT |
| GS-F | CCGTTCTACGAGGAATCGAC |
| GS-R | GACATGTCCGTGTTCCACTG |
| OTC-F | GGCGGACATCTTCACGTACT |
| OTC-R | GGATCCTCGACCACTTCGTA |
| AGK-F | GCAAGACCGTGGTGATCAAG |
| AGK-R | CTCGACGACTTCCATGGTTT |
| AS-F | CCGATGTTCAACCTGCATC |
| AS-R | CATCGAAGCTGGTCAGGTG |
| 16S rRNA-F | TCTGAGAGGACGACCAGCCA |
| 16S rRNA-R | CGAAGGCCTTCTTCACACAC |
| PII-F | CAGCATCACCTGACGATGAG |
| PII-R | CGGTAGAGGTCTTCCAGCAG |
| AmtB-F | CCTGGCTCAAGATCAAGCTC |
| AmtB-R | TCAGGATGATGAAGGTCACG |
| PmaCI-F | CACGTGCTCGAGAACGTCTATATCAAGGCC |
| PmaCI-R | CACGTGTTAAAAGTTGTACTCCAGCTTGTG |
| Tn3gus | CCGGTCATCTGAGACCATTAAAAGA |

F- forward primer; R – reverse primer

**Table S3.** Differentially expressed genes in *grtK* and *grtR* mutants compared to the wild type

| Regulation | Function^a^ (amount of genes) | | Regulation | Function^a^ (amount of genes) | | Regulation | Function^a^ (amount of genes) | |
| --- | --- | --- | --- | --- | --- | --- | --- | --- |
| Up-regulated genes in  wild type  (activated by GrtK/R) | **Ammonium assimilation** | **3** | Down-regulated genes in  *grtK* mutant  (derepressed by GrtK) | **Ammonium assimilation** | **1** | Down-regulated genes in  *grtR* mutant  (derepressed by GrtR) | Amino acid metabolism | 3 |
|  | **Ammonia detoxification** | **2** |  | Carbohydrate metabolism | 3 |  | 2nd metabolite synthesis | 1 |
|  | **Oxalate biosynthesis** | **2** |  | Amino acid metabolism | 4 |  | Coenzyme metabolism | 3 |
|  | Amino acid metabolism | 17 |  | Amino acid synthesis | 1 |  | Oxidoreductase, Electron transport | 4 |
|  | Fatty acid metabolism | 12 |  | Fatty acid metabolism | 5 |  | Signal transduction | 2 |
|  | Lipid biosynthesis | 3 |  | Coenzyme metabolism | 5 |  | Nucleotide metabolism | 1 |
|  | ATP synthase | 5 |  | Oxidoreductase, Electron transport | 10 |  | Nucleotide synthesis | 1 |
|  | Oxidoreductase, Electron transport | 12 |  | Inorganic transport | 3 |  | DNA replication | 1 |
|  | Hydroperoxide reductase | 1 |  | Protein transport | 2 |  | Cell membrane biogenesis | 3 |
|  | Signal transduction | 4 |  | Cell membrane biogenesis | 5 |  | Cell division | 3 |
|  | Inorganic transport | 2 |  | Transcription regulator | 6 |  | Transcription | 3 |
|  | Nucleotide metabolism | 4 |  | Translation | 1 |  | Transcription regulator | 6 |
|  | DNA replication | 2 |  | Ribosomal protein | 1 |  | Translation | 3 |
|  | Cell membrane biogenesis | 17 |  | Hypothetical protein | 12 |  | Molecular chaperone | 7 |
|  | Cell division | 1 |  | Unknown function | 25 |  | Ribosomal protein | 5 |
|  | Transcription | 4 |  |  |  |  | Hypothetical protein | 6 |
|  | Transcription regulator | 4 |  |  |  |  | Unknown function | 18 |
|  | Translation | 10 |  |  |  |  |  |  |
|  | Ribosomal protein | 18 |  |  |  |  |  |  |
|  | Hypothetical protein | 19 |  |  |  |  |  |  |
|  | Unknown function | 36 |  |  |  |  |  |  |
|  | Total | 178 |  | Total | 84 |  | Total | 70 |
| Down-regulated genes in  wild type  (repressed by GrtK/R) | Amino acid metabolism | 1 | Up-regulated genes in  *grtK* mutant  (repressed by GrtK) | Fatty acid metabolism | 2 | Up-regulated genes in  *grtR* mutant  (repressed by GrtR) | Coenzyme metabolism | 1 |
|  | 2nd metabolite synthesis | 3 |  | Coenzyme metabolism | 1 |  | Inorganic transport | 2 |
|  | Cell cycle control | 2 |  | Signal transduction | 1 |  | DNA repair | 2 |
|  | Transcription | 2 |  | Cell cycle control | 2 |  | Transcription | 2 |
|  | Transcription regulator | **1** |  | Transcription | 4 |  | Transcription regulator | 2 |
|  | Hypothetical protein | 9 |  | Transcription regulator | 2 |  | Hypothetical protein | 3 |
|  | Unknown function | 6 |  | Translation | 1 |  | Unknown function | 9 |
|  |  |  |  | Molecular chaperone | 4 |  |  |  |
|  |  |  |  | Flagellar (FliO) | 1 |  |  |  |
|  |  |  |  | Hypothetical protein | 8 |  |  |  |
|  |  |  |  | Unknown function | 24 |  |  |  |
|  | Total | 24 |  | Total | 50 |  | Total | 21 |

(a) Groups related by function of genes are eggNOG (evolutionary genealogy of gene: Non-supervised Orthologous Groups) database (7), and related metabolic pathways are KEGG (Kyoto Encyclopedia of Genes and Genomes) database (8). Bold type indicates genes related to ammonia metabolism.

**Table S4.** Key genes differentially expressed in *grtK* or *grtR* mutants

| GeneID^a^ (gene) | Transcriptome expression^b^ | | | edgeR  p-value^c^ | Contig^d^ | Product / Function |
| --- | --- | --- | --- | --- | --- | --- |
|  | wild type | *grtK* | *grtR* |  |  |  |
| **Differentially expressed genes activated or derepressed by GrtK or GrtR** | | | | | | |
| bglu_1g05580 | 418.8688 | 220.7822 | 159.7047 | 0.016 | Chr. 1 | glutamate dehydrogenase (GDH) / ammonia assimilation |
| bglu_1g05590 | 209.7817 | 34.3439 | 13.4206 | 0.000 | Chr. 1 | glutamate/aspartate ABC transporter / uptake of L-glutamate and L-aspartate |
| bglu_1g25000 (*glnA*) | 676.5808 | 40.4767 | 37.5776 | 0.000 | Chr. 1 | glutamine synthetase (GS) / ammonia assimilation and detoxification of ammonia |
| bglu_1g29180 (*argF*) | 166.714 | 60.1018 | 72.471 | 0.040 | Chr. 1 | ornithine transcarbamylase / facilitates steps to capture toxic ammonia and transform it into urea |
| bglu_1g33800 (*argB*) | 31.9535 | 8.586 | 16.1047 | 0.038 | Chr. 1 | acetylglutamate kinase **/** facilitates steps to capture toxic ammonia and transform it into urea |
| bglu_2g15870 (*rpoD*) | 236.1781 | 93.2191 | 29.5252 | 0 | Chr. 2 | RNA polymerase sigma factor RpoD (sigma 70) / principal sigma factor responsible for the transcription of housekeeping genes in most bacteria |
| bglu_2g18790 (*obcA*) | 1682.4217 | 339.7592 | 185.2038 | 0.001 | Chr. 2 | 3-keto-5-aminohexanoate cleavage protein / oxalate biosynthesis |
| bglu_2g18780 (*obcB*) | 256.3227 | 104.2582 | 60.3925 | 0.001 | Chr. 2 | γ-carbonic anhydrase / oxalate biosynthesis |
| bglu_2g06750 | 33.3428 | 0.0 | 53.6823 | 0.000 | Chr. 2 | asparagine synthase / assimilation of nitrate or ammonium into asparagine for use in nitrogen recycling. |
| **Differentially expressed genes repressed by GrtK or GrtR** | | | | | | |
| bglu_1g12390 | 0.6946 | 11.0391 | 5.3682 | 0.006 | Chr. 1 | threo-3-hydroxy-D-aspartate ammonia-lyase / It catalyzes the conversion of threo-3-hydroxy-D-aspartate to oxaloacetate and ammonia. |
| bglu_1g06340 (*dnaK*) | 2963.3407 | 8612.9576 | 977.0169 | 0.020 | Chr. 1 | 70 kilodalton heat shock proteins (DnaK) / molecular chaperone |
| bglu_1g07140 | 21.5339 | 62.555 | 6.7103 | 0.045 | Chr. 1 | co-chaperone GroES / molecular chaperone |
| bglu_1g31450 (*rpoH*) | 315.3672 | 989.84 | 76.4972 | 0.014 | Chr. 1 | RNA polymerase sigma factor RpoH / the key regulator of the heat shock response in *Escherichia coli* |
| bglu_1g26820 (*htpG*) | 539.7365 | 1333.279 | 241.5701 | 0.048 | Chr. 1 | molecular chaperone HtpG / molecular chaperone |
| bglu_2g19340 | 1.3893 | 11.0391 | 1.3421 | 0.028 | Chr. 2 | co-chaperone GroES / molecular chaperone |
| bglu_1p1190 | 12.5036 | 14.7188 | 36.2355 | 0.032 | Pla. 1 | sigma-54-dependent family transcriptional regulator / modulated in response to to chemical and metabolic changes are responsible for determining the level of expression of sigma 54-dependent genes |

(a) Gene IDs were obtained from the *B. glumae* BGR1 genome database (GenBank accession numbers: CP001503–CP001508).

(b) The Transcriptome expression represents Reads Per Kilobase per Million mapped reads (RPKM). RPKM = gene mapped reads / (total mapped reads (millions) × gene length (Kb))

(c) Differential gene expression analysis based on the empirical bayes estimation (edgeR) (9). Genes shown in the table are selected with p-value of 0.05 or less.

(d) Chr. 1, Chr. 2, and Pla. 1 means Chromosome 1 (https://www.ncbi.nlm.nih.gov/nuccore/CP001503.2), Chromosome 2 (https://www.ncbi.nlm.nih.gov/nuccore/CP001504.2), and Plasmid 1 (https://www.ncbi.nlm.nih.gov/nuccore/CP001505.1) of *B. glumae* BGR1 respectively.

**Fig. S1**

Ammonia accumulation of *B. glumae* strains was measured in each medium. The ammonia concentrations of the *B. glumae* wild-type strain BGR1, BGRK1 (BGR1 *grtK*::Tn*3-gusA24*) and BGRR1 (BGR1 *grtR*::Tn*3-gusA54*) with pLAFR3 (empty vector), and the *grtK* and *grtR* mutants complemented with pGRT2 were determined in LB, HEPES-buffered LB (pH 7), KB, and M9 media. Error bars indicate the standard error (SE) ranges from three independent experiments.

**Fig. S2**

Expression levels of the *qsmR* gene, a quorum sensing (QS) master regulator, were examined in GrtK/R TCS mutants. Quantitative reverse transcription PCR (qRT-PCR) was used to quantify gene expression levels in the wild-type strain BGR1, BGRK1 (BGR1, *grtK*::Tn*3-gusA24*), and BGRR1 (BGR1, *grtR*::Tn*3-gusA54*) after 12 h of incubation in LB and M9 media, with three biological replicates.

**Fig. S3**

The expression levels of the P_II_ protein gene (bglu_1g12700; P_II_ protein gene and *amtB* (bglu_1g31870; the ammonium transporter of *B. glumae* BGR1), were examined in GrtK/R TCS mutants. Using quantitative reverse transcription polymerase chain reaction (qRT-PCR), the expression levels of these genes were quantified in the wild-type strain BGR1, BGRK1 (BGR1, *grtK*::Tn*3-gusA24*), and BGRR1 (BGR1, *grtR*::Tn*3-gusA54*) in both LB and M9 media after 12 h of incubation, with three biological replicates. The bars indicate ± SE. Asterisks (*) denote a significant difference (p < 0.05) in the expression level between the mutant and the wild-type strain based on ANOVA/Tukey’s correction for multiple comparisons.

**Fig. S4**

L E N V Y I K A D K Q K N G I K A N F K

1 CTCGAGAACGTCTATATCAAGGCCGACAAGCAGAAGAACGGCATCAAGGCGAACTTCAAG 60

I R H N I E D G G V Q L A Y H Y Q Q N T

61 ATCCGCCACAACATCGAGGACGGCGGCGTGCAGCTCGCCTACCACTACCAGCAGAACACC 120

P I G D G P V L L P D N H Y L S V Q S K

121 CCCATCGGCGACGGCCCCGTGCTGCTGCCCGACAACCACTACCTGAGCGTCCAGTCCAAG 180

L S K D P N E K R D H M V L L E F V T A

181 CTGAGCAAAGACCCCAACGAGAAGCGCGATCACATGGTCCTGCTGGAGTTCGTGACCGCC 240

A G I T L G M D E L Y K G G T G G S M V

241 GCCGGGATCACTCTCGGCATGGACGAGCTGTACAAGGGTGGTACCGGTGGATCTATGGTG 300

S K G E E L F T G V V P I L V E L D G D

301 AGCAAGGGCGAGGAGCTGTTCACCGGGGTGGTGCCCATCCTGGTCGAGCTGGACGGCGAC 360

V N G H K F S V S G E G E G D A T Y G K

361 GTAAACGGCCACAAGTTCAGCGTGTCCGGCGAGGGCGAGGGCGATGCCACCTACGGCAAG 420

L T L K F I C T T G K L P V P W P T L V

421 CTGACCCTGAAGTTCATCTGCACCACCGGCAAGCTGCCCGTGCCCTGGCCCACCCTCGTG 480

T T L T Y G V Q C F S R Y P D H M K Q H

481 ACCACCCTGACCTACGGCGTGCAGTGCTTCAGCCGCTACCCCGACCACATGAAGCAGCAC 540

D F F K S A M P E G Y I Q E R T I F F K

541 GACTTCTTCAAGTCCGCCATGCCCGAAGGCTACATCCAGGAGCGCACCATCTTCTTCAAG 600

D D G N Y K T R A E V K F E G D T L V N

601 GACGACGGCAACTACAAGACCCGCGCCGAGGTGAAGTTCGAGGGCGACACCCTGGTGAAC 660

R I E L K G I D F K E D G N I L G H K L

661 CGCATCGAGCTGAAGGGCATCGACTTCAAGGAGGACGGCAACATCCTGGGGCACAAGCTG 720

E Y N F N

721 GAGTACAACTTTTAA

The DNA sequence of mcpGFP used in this study includes synthetic linker segments, which are highlighted in yellow.

**Fig. S5**

Southern blot analysis was employed to validate the marker-exchanges in (A) BGRK1 (BGR1, *grtK*::Tn*3-gusA24*) and (B) BGRR1 (BGR1, *grtR*::Tn*3-gusA54*). Southern hybridizations were performed on 1 μg of DNA digested using the *Bam*HI restriction enzyme and subsequently hybridized with a pGRT1 probe. The numbers on the left side of the blot indicate DNA molecular markers, measured in kilobases (Kb). (A) Lane 1: Kb marker, Lane 2: pGRT1, Lane 3: BGR1, Lane 4: BGS9, Lane 5: pGRT1 (*grtK*::Tn*3-gusA24*), Lane 6: BGR1 (*grtK*::Tn*3-gusA24*), Lane 7: pGRT1 (*grtK*::Tn*3-gusA5*), Lane 8: BGR1 (*grtK*::Tn*3-gusA5*). (B) Lane 1: Kb marker, Lane 2: pGRT1, Lane 3: pGRT1 (*grtR*::Tn*3-gusA54*), Lane 4: BGR1, Lane 5: BGR1 (*grtK*::Tn*3-gusA54*).

**REFERENCES**

1. Choi KH, DeShazer D, Schweizer HP. 2006. mini-Tn 7 insertion in bacteria with multiple *glmS*-linked att Tn 7 sites: example *Burkholderia mallei* ATCC 23344. Nat Protoc 1**:**162–169.

2. Kim J, Kim JG, Kang Y, Jang JY, Jog GJ, Lim JY, Kim S, Suga H, Nagamatsu T, Hwang I. 2004. Quorum sensing and the LysR-type transcriptional activator ToxR regulate toxoflavin biosynthesis and transport in *Burkholderia glumae*. Mol Microbiol 54:921–934.

3. Kim J, Kang Y, Choi O, Jeong Y, Jeong JE, Lim JY, Kim M, Moon JS, Suga H, Hwang I. 2007. Regulation of polar flagellum genes is mediated by quorum sensing and FlhDC in *Burkholderia glumae*. Mol Microbiol 64:165–179.

4. Staskawicz B, Dahlbeck D, Keen N, Napoli C. 1987. Molecular characterization of cloned avirulence genes from race 0 and race 1 of *Pseudomonas syringae* pv. *glycinea*. J Bacteriol 169**:**5789-5794.

5. Keen NT, Tamaki S, Kobayashi D, Trollinger D. 1988. Improved broad-host-range plasmid for DNA cloning in gram negative bacteria. Gene 70:191–197.

6. Marunga J, Goo E, Kang Y, Hwang I. 2021. Identification of a Genetically Linked but Functionally Independent Two-Component System Important for Cell Division of the Rice Pathogen *Burkholderia glumae*. Front Microbiol 12:700333. doi: 10.3389/fmicb.2021.700333.

7. Huerta-Cepas J, Szklarczyk D, Heller D, Hernández-Plaza A, Forslund SK, Cook H, Mende DR, Letunic I, Rattei T, Jensen LJ, von Mering C, Bork P. 2019. eggNOG 5.0: a hierarchical, functionally and phylogenetically annotated orthology resource based on 5090 organisms and 2502 viruses. Nucleic Acids Res 47(D1):D309–314.

8. Kanehisa M, Sato Y, Kawashima M, Furumichi M, Tanabe M. 2016. KEGG as a reference resource for gene and protein annotation. Nucleic Acids Res 44(D1):D457–462.

9. Robinson MD, McCarthy DJ, Smyth GK. 2010. edgeR: a Bioconductor package for differential expression analysis of digital gene expression data. Bioinformatics 26:139–140.
